# 

**Published:** 1891-03-14

**Authors:** 


### GENERAL CHARITIES.

[This Column is devoted to an account of work of charitable institutions other than Medical Charities. The Editor will be glad to receive reports or news of work at 140, Strand. *Philanthropy* should be plainly written on the envelope.]

We regret to hear that such a useful institution as **THE ROYAL ALBERT ORPHAN ASYLUM** at Bagshot should have suffered such a considerable falling off in receipts lately, as compared with the average of the previous seven years, that the management felt compelled to withold the usual election last November for want of funds. For the same reason 50 of the 220 beds which the asylum holds for boys and girls are now vacant. These facts are the more regretable as the institution is carried on upon the *non-canvassing* system with a view to enabling the really friendless and destitute children to compete with candidates at other asylums where influence and means have greater consideration than merit or need. Bagshot being one of the healthiest localities in England it is not surprising to find that the health of the children is, and always has been good, and any visitors from London will speedily notice the difference between the bright ruddy faces of the orphans and the pale wan look of London children. The girls are trained to become good and useful servants, and the boys, who do the work of the kitchen-garden and farm, are taught carpentering and painting. The elder boys are taught fire drill, and a post in the Camberley Fire Brigade is coveted as one of trust and reward. The finances of the Institution are heavily handicapped by a mortgage of £12,500 with an annual charge of £520 for interest. If this mortgage can be extinguished a reduction in the number of children will not be necessary. Donations for the purpose of enabling the management to pay off the burden, and for the general support of the Institution will be gratefully acknowledged by the Secretary, Mr. H. W. Tatum, 62, King William Street, E.C.

The **HOMES FOR LITTLE BOYS** at Farningham and Swanley are, we believe, the prototypes of many useful institutions in this country. The cottage system originated at Farningham has been so successful that it has not only been imitated by other charitable societies, but also by Poor Law Guardians. Though the principles of the two Homes are identical the objects are somewhat dissimilar. That of the Cottage Homes at Farningham is to feed, clothe, educate, and train to industrial work homeless and destitute little boys and those in danger of falling into crime, whether orphans or not, who are disqualified by poverty or other circumstances for admission to orphan asylums or other institutions. All such children must be under ten years of age at the time of admission. The Homes at Swanley, in contradistinction, are designed for orphan and fatherless boys, who might otherwise be eligible for election to orphan asylums but whose relatives and friends are desirous of doing what is in their power towards their support, and who agree to contribute the yearly sum of £20 towards their maintenance. The mode of admission is solely by payment of this sum, and boys are received at Swanley at any age up to twelve, being retained till they are fit to go out into the world and earn their own livelihood. The mode of admission to the Cottage Homes at Farningham is twofold. One, by election of the subscribers. Boys thus admitted are received free, boarded, clothed, educated, and taught some industrial occupation, until they are fourteen and a-half years of age. Secondly, by payment of six shillings a week, paid quarterly in advance, or of £15 a year paid in one sum. Those who are received on payment are admitted as soon as a vacancy arises, and thus urgent cases can be attended to without waiting for election, which only takes place in the months of June and December. The absence of the system of voting, the objections to which we have endeavoured previously to point out, in the case of the Swanley Homes, is a recommendation to many people. The Committee must be better able to judge of the merits of the cases which come before them than the subscribers, and we are glad to learn that the feelings of the majority of the Committee are so strongly opposed to this objectionable system that in the endeavour, so far as possible, to do away with it, they invite subscribers to place votes at their disposal. That they will ever succeed in getting all the subscribers to divest themselves of their little brief authority is perhaps hopeless to expect. It is a fundamental principle of both Homes that the education imparted to the children shall be unsectarian; thus restrictive principles and bitterness of party spirit are avoided. There are now eleven cottages, built to accommodate thirty boys each, presided over by a man and his wife who act as father and mother to the boys. Every endeavour is made to make the cottages homes in reality. When the children have reached a certain standard, the fourth, in their schooling, they are allowed to go into the workshops, and become half-timers until they pass the sixth standard, after which they go to work all day and to school in the evening. Thus they learn a trade and how to make a living when they leave. All the shoes and clothes are made on the premises, and all the carpentry is also done there. The state of the finances of the institutions is not quite what their friends would wish to see. For many years the income has not equalled the expenditure, and the Committee rely on extraordinary measures, such as the annual dinner, for providing the necessary funds to maintain the 300 boys at Farningham, and the 200 at Swanley. It appears that for some three years two of the houses at the former place have been closed owing to this reason, though only one, we understand, is tenantless at the present moment. During the holidays the boys do not leave the Homes, so that any donations to help the management to give them a day at the sea-side, as well as donations for the general purposes of the Institution will be gratefully received by the Secretary, Mr. Benjamin Clarke, Bank Buildings, Ludgate Circus, E.C.

### SCRAPS AND GLEANINGS.

THE Convalescent Home for Men, for providing which Colonel Seeley has offered £10,000, will be at Skegness.

THE annual conversazione in aid of the Ballymena Cottage Hospital was held in Ballymena on February 19th.

AN Italian bazaar, in aid of the Italian Benevolent Fund, was opened at St. Stephen's Hall, on Monday, March 9th.

IT is proposed to celebrate the centenary of the Belfast Royal Hospital by having a bazaar in aid of the funds of the hospital.

THE total receipts for 1890 of the Bradford Eye and Ear Hospital amounted to £2,197, and the expenditure for the year was £1,982.

DURING the ensuing month the Royal Academy propose to fill up the professorship of anatomy, rendered vacant by the death of Professor Marshall.

THE authorities of the Paddington Green Children's Hospital are making an effort to enlarge their building in order to provide a few more permanent beds.

THE Prince of Wales will preside at a festival dinner on behalf of the Eastern Counties Asylum for Idiots, Colchester, at the Hotel Metropole, on Friday, April 24th.

THE Lord Mayor and Sheriffs will attend the dinner at the Hotel Metropole on March 18th, in aid of the Hospital for Sick Children. The Duke of Fife will take the chair.

PROFESSOR BILLROTH, of Venice, has cured two patients with Dr. Koch's remedy—one of lupus of the nose and upper lip, the other of actinomycos of the abdominal wall.

THE Royal Commission on Vaccination held another meeting on the 4th inst., under the presidency of Lord Herschell, and received a deputation of anti-vaccinationists from Leicester.

THE Committee of the Southampton Hospital Sunday Fund report that last year the result of the collections was £1,183 7s., this being the largest total yet attained in Southampton.

DR. ROBERTS has resigned his position in the Keighley Cottage Hospital, as his five colleagues declined to co-operate with him, on the ground that he used Count Mattei's remedies for cancer.

AN old man, named James Grimm, attempted to crucify himself at Philadelphia last week. He marked a rough cross on the floor, and nailed one hand and his feet to the boards, but was then discovered.

MRS. CALVERLEY BEWICKE'S recitations on March 3rd, at the Westminster Town Hall, were most successful. The room was crowded, and the Samaritan Fund of the Westminster Hospital will be the richer by £55.

THE Sub-Committee appointed to look into the accounts of the Cardiff Infirmary report that the regular annual subscriptions must be increased by at least £1,000 if the present work of the Infirmary is to be carried on efficiently.

AN alarming outbreak of pneumonia among children has occurred at Rounds, Northamptonshire. The children range from four to seven years of age. There are nearly 70 serious cases, and three deaths have already occurred.

THE sixty-third annual report of the Sussex County Hospital shows a deficit on the past year's accounts of more than £1,000. The governors have been compelled to sell stock to the value of £500, and still start the present year with a deficiency of a similar sum.

A CONCERT was given on Saturday, March 7th, at the St. James's Hall, in aid of the proposed Caxton wing of the Morley House Seaside Convalescent Home for Working Men, St. Margaret's Bay. The Lord Mayor and Sheriffs were present.

"ONE OF THE MEDICAL STAFF" writes: Many thanks for the note on the North-West London Hospital. It ought to do us some good. In fact the secretary has already received a cheque for £50 from a lady at Brighton who reads THE HOSPITAL.

THE Queen has sent two photogravures of the Jubilee portrait of herself to the leper settlement on Robben Island. They bear the inscription: "Presented by her Majesty the Queen to her suffering subjects in the Leper Hospital, Robben Island, 1891."

AT a recent meeting of the Hospital Aid Society, at Fleetwood, the following resolution was moved and carried unanimously: "That in the opinion of this meeting, the time has arrived when a united endeavour should be made to secure suitable premises for the purpose of a cottage hospital in the town."

THE annual meeting of the subscribers to the Royal Portsmouth, Portsea, and Gosport Hospital was held in the Grand Jury Room at the Town Hall, Portsmouth. Sir William King read the forty-second annual report of the Committee of Management. It stated that a total of 937 in-patients received treatment. The total number of out-patients during the year was 7,915, and of that number 2,380 who had met with casualties were treated without a subscriber's recommendation. The balance sheet showed a total expenditure of £6,376, and an income of £6,056.

ON February 23rd, Sir William Evans presided over a specially-called meeting of the Governors of the Derbyshire General Infirmary, to consider the plans prepared by the architect for the erection of the proposed new infirmary. Mr. Young, one of the architects, gave a description of the proposed new buildings from the plans, the cost of erecting which is roughly estimated at £[illegible]4,000. The following was moved and carried: "That the Building Committee be requested to form at once for itself an Infirmary Appeal Committee, who shall draw up an appeal for funds with such plans and information as shall be deemed necessary."

THE Senate of the University of London has received communications from the Royal College of Physicians and the Royal College of Surgeons to the effect that both these institutions had given their complete adherence to the scheme proposed for granting a medical degree to London students after going through a prescribed course of education and passing the required examination by a competent board, formed by representatives from the University and the two Royal Colleges. This will be an immense boon to the students of the London Medical Schools who have hitherto been obliged to go in search of degrees bestowed by foreign or provincial Universities.

### Books Received.

Cassell and Co.

"The Year Book of Treatment for 1891." Price 7s. 6d.

Cornish Bros. (Birmingham).

"The Elements of Ophthalmic Therapeutics." D. C. Lloyd-Owen F.R.C.S.I.

### THE STUDENT OF MEDICINE.

[All communications for this Supplement should be addressed to The Editor of The Hospital, at 140, Strand, with the words, "Students' Column," written in the left hand top corner of the envelope.]

LEADING ATHLETES.

The portrait we give this week is that of Mr. B. C. Green, of St. Bartholomew's, the Secretary of the United Hospitals Athletic Club. He was educated first at Clewer House School, Windsor, and then went to Bedford Grammar School. For many years he went in a good deal for athletic exercises, though not of a public character. He joined St. Bartholomew's Hospital in 1888, and up to that time he had confined himself almost entirely to high jumping, and in a small degree to cross-country running after foxhounds. His best jump at high jumping has been 5 ft. 10 in., and in this branch he has carried off a large number of prizes. Shortly after coming to London he joined the Paddington Recreation Ground Athletic Club, for the purpose of high-jump practice, and while there he discovered that he possessed the necessary qualities of a "sprinter," and determined to try his luck on the "path." His first race was at the Blackheath Harriers meeting at Catford Bridge in July, 1888, where he won the 100 yards from the 7½ yards mark in very easy style. After that event handicappers began to look after him, and he soon found himself getting nearer and nearer the "scratch" mark.

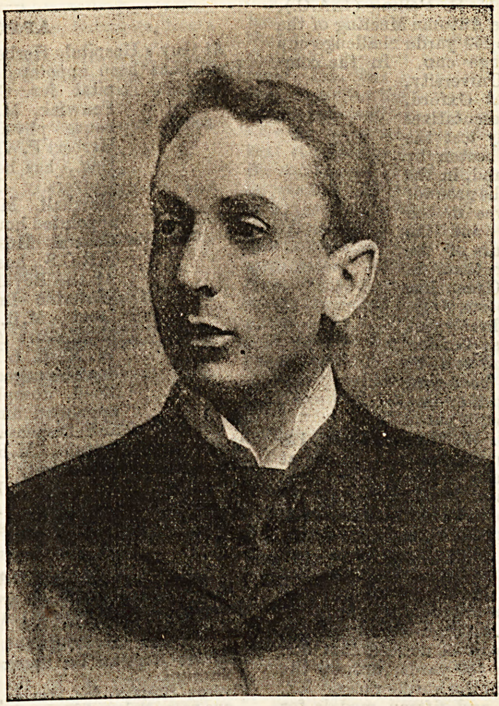

*From a photograph by Walery.*
MR. B. C. GREEN (St. Bartholomew's).

He joined St. Bartholomew's too late in 1888 to compete in the Inter-Hospital Sports that year, but in 1889 he represented his hospital as "second string" in the 100 yards and high jump, but only obtained third place in each event. In July of the same year he ran for the United Hospitals in the first meeting with Edinburgh University, at Edinburgh, and tied the high jump with the Edinburgh representative at 5 ft. 6 in., while in the 100 yards he ran third to a dead heat between G. S. S. Marshall (U.H.A.C.) and R. A. Taylor (E.U.A.C.). This year he improved considerably in his running, and at the United Hospitals Sports, on July 1st, he was successful in placing both the 100 yards and quarter-mile challenge cups, the 220 yards and the 120 yards hurdle race to the credit of St. Bartholomew's, a performance which has never been surpassed and only once equalled in the annals of the Hospital Athletics, when in 1867 G. R. Nunn, of Guy's Hospital, won four events. In this year's competition with Edinburgh, then at Stamford Bridge, the Hospitals were even more victorious than the previous year, winning nine out of the ten events, three of which, namely, the 100 yards, 440 yards, and

THE STUDENT OF MEDICINE—(*continued*).

120 yards hurdle race, Mr. Green carried off. In the first meeting of the Hospitals with the London Athletic Club, which was held at Stamford Bridge on July 26th of this year, the 100 yards resulted in a dead heat between three, viz., E. H. Pelling (L.A.C.), Marshall and Green (U.H.A.C.). In the Autumn Meeting of the L.A.C. on October 6th, he carried off the 250 yards challenge cup and the 440 yards hurdle race challenge cup. In the competition between the L.A.C. and Oxford University, which was held in November, 1890, at Oxford, he defeated both the University representatives in the 120 yards hurdle race, and only lost the first place by a few inches, being beaten by C. W. Haward, late champion of England, in the good time, considering the wet state of the ground, of 16 4-5 sec. His latest performance was in the match between Cambridge University and the L.A.C., when, running under the colours of the latter club, he secured the victory for them in the hurdles. In open handicaps Mr. Green has done as follows: Sydenham athletic sports, July, 1889, he won the 100 yards from the three yards mark in 10 2-5 sec., also the 300 yards; London Athletic Club sports, 1890, he ran second to J. P. Shuter (L.A.C.) in the440 yards, being beaten on the tape for first place in 51 4-5 sec. On the same day he ran second to E. H. Pelling in the 100 yards challenge cup race, and also second to C. W. Haward in the 120 yards hurdle challenge cup. In the Kensington A.A. sports at Paddington he obtained the second place in the 120 yards in 12 2-5 sec. In the Civil Service sports at Stamford Bridge he was beaten on the tape for the first place in the 120 yards hurdle race by G. Shaw, hurdle race champion of New Zealand. In the Scottish gathering of 1888 at Stamford Bridge he won the open high jump and 100 yards (Scotsmen only), and in the same meeting of 1889 the high jump and 100 yards. In the Stoke-on Trent athletic sports in August, 1890, he won the open 440 yards, receiving one yard from H. R. Hooper (L.A.C.), in 50 2-5 sec. In reviewing this long list of successes it will be noticed that Mr. Green's best distances are from 220 to 440 yards, but, on the whole, his best performances have been in the hurdle races. Mr. Green now holds four challenge cups and fifteen medals for "scratch" races, together with from seventy to eighty other prizes. He is also a good fencer and all-round gymnast, and an excellent shot.

### APPOINTMENTS.

At Guy's Hospital, Herbert V. Hickman, M.R.C.S., L.R.C.P. Lond., has been appointed ophthalmic assistant. At the Hospital for Consumption, Brompton, W. Soltau Fenwick, M.D. B.S. Lond., has been appointed house physician. At Charing Cross Hospital, Herbert F. Waterhouse, M.D., C.M. Edin., F.R.C.S. Eng., has been appointed assistant surgeon. At University College Hospital, Charles Devereux Marshall, M.R.C.S., L.R.C.P. Lond., has been appointed house surgeon. At the Great Northern Central Hospital, Holloway Road, W. Adams, F.R.C.S., has been appointed consulting surgeon. At the Royal Hospital for Children and Women Waterloo Bridge Road, S. W. Wheaton, M.D. Lond., M.R.C.P., D.P.H., has been appointed physician. At the Metropolitan Hospital, Sibley W. Read, L.D.S.R.C.S. Eng., has been appointed dental surgeon. At St. Mark's Hospital, City Road, Joseph Mills, M.R.C.S., &c., has been appointed anæsthetist. At St. Alban's Hospital and Dispensary, Eustace H. Lipscomb, B.A., M.B., B.C. Cantab., M.R.C.S. Eng., L.R.C.P. Lond., has been appointed honorary medical officer. At the Doncaster Infirmary, F. Webb, L.R.C.P.Lond., M.R.C.S., has been appointed house surgeon. At Lynton, F. C. Berry, M.D., M.B., B.Ch., has been re-appointed medical officer of health.

### VACANCIES.

The following vacancies are announced this week, the amount of salary in each case being given after the name of the institution:

### Resident Medical Officers.

Belgrave Hospital for Children, 79, Gloucester Street, S.W.—House Surgeon.

Chelsea Hospital for Women, Fulham Road, S.W., Medical Officer for one year—Salary £80, &c.

### THE STUDENT OF MEDICINE—(*continued*).

Leith Hospital, House Physician and House Surgeon—Salary £50 each.

Liverpool Northern Hospital, Assistant House Surgeon—Salary £70, &c.

North-Eastern Hospital for Children, Hackney Road, N.E., Junior House Surgeon—Salary £5 a month.

Royal Free Hospital, Gray's Inn Road, Junior Resident Medical Officer.

Royal Bath Hospital and Rawson Convalescent Home, Harrogate, House Surgeon and Secretary—Salary £80, &c.

St. Peter's Hospital for Stone and Urinary Diseases, Henrietta Street, Covent Garden. House Surgeon—Honorarium 25 guineas, &c.

Sussex County Asylum, Haywards Heath, Junior Assistant Medical Officer—Salary £100, &c.

Teignmouth, Dawlish, and Newton Infirmary and Convalescent Home, House Surgeon (to act also as Secretary)—Salary £40.

West Bromwich District Hospital, Assistant House Surgeon.

West Ham Union, Assistant Medical Officer—Salary £100, &c.

West London Hospital, Hammersmith Road, House Physician and House Surgeon.

Rotherham Hospital, Assistant House Surgeon.

St. John's Hospital for Diseases of the Skin, Leicester Square, W.C., House Surgeon.

#### Non Resident Medical Officers.

St. George's and St. James's Dispensary, King's Street, Regent Street, Surgeon.

Dental Hospital of London, Four Demonstraters—Honorarium £50.

Hospital for Women, Soho Square, W., Clinical Assistants to out-patient department.

Hull Royal Infirmary, four Honorary Assistant Surgeons and Ophthalmic Surgeon.

King's College, London, Demonstrator of Physiology.

Norfolk and Norwich Hospital, Physician on the Honorary Medical Staff.

St. Pancras and Northern Dispensary, 126, Euston Road, Honorary Physician.

Turner's Hospital, Kirkleatham, Surgeon.

### STUDENTS' MEDICAL SOCIETIES.

#### Guy's Hospital Pupils' Physical Society.

A meeting of this society was held in the Chemical Theatre on Saturday, January 24th, Mr. F. G. Hopkins in the chair, and 38 members being present. Mr. A. M. Daldy read a paper on "Cold in the Causation of Disease, and Ice as a Therapeutic Agent." After enumerating a large number of diseases commonly attributed to "cold," amongst which diseases of the respiratory organs had the preponderance, Mr. Daldy remarked that nine out of every ten cases of the latter were bronchitis. As a general classification of the diseases attributed to cold, he would divide them into (a) diseases characterised by inflammation and congestion, and (b) functional nervous diseases. There was only one disease, in the causation of which cold was an important factor, which could not be brought under one of these heads, and that was progressive muscular atrophy. His paper went to show that cold was not the exciting cause for nephritis, rheumatic fever, &c., but a predisposing cause, which made conditions favourable for the action of the specific cause (whatever it was), which was uniform and constant for any given disease. Considering ice as a therapeutic agent, the conclusion seemed to be that it might be a valuable remedy for inflammation, so long as the condition had not advanced to the stage when resolution was impossible. Of 42 cases of pneumonia treated with the icebag, in 60 per cent. a favourable result had followed, in 25 per cent. no result, and in 15 per cent. an unfavourable result, amongst the last being four cases in which collapse had resulted from the application. With reference to cold in nosology, he would make three classes of diseases: (1) Those formerly attributed to cold, of which the definite cause was now known, viz., the specific fevers, phthisis, &c.; (2) Those which are still allowed to be caused by cold, such as myelitis; and (3) those still under discussion, as pneumonia and diphtheria. Mr. A. S. Wohlmann opened the discussion, which was continued by Messrs. T. R. Taylor, E. T. E. Hamilton, Guy Mackeson, A. J. Sharp, A. T. Rake, and F. M. Turner. Mr. Daldy replied in detail to the questions and criticisms of the speakers.

#### Royal Veterinary College.

The 141st ordinary general meeting of the Royal Veterinary College Medical Association was held in the theatre of the College on Wednesday, January 28th, Mr. G. H. Williams, Vice-President, in the chair. There were present Professors Axe and Penberthy, Messrs. Coghill and Edwards, 23 student members, three visitors, and the Assistant Secretary. The minutes of the last meeting were read and confirmed. Mr. Coghill read a paper on "Microtomes Section—Cutting and Mounting." He stated that fifty or sixty years ago the method of preparing sections as we now know it was unknown, but that the method of preparation was that of teazing out tissues, and in this manner a perfection was obtained which we at the present time

### THE STUDENT OF MEDICINE—(*continued*).

could form little idea of. He mentioned that as far back as 1770 various forms of microtomes were in use for the cutting of hard structures, such as wood and horn, but it was not until 1857 that microtomes were used for the cutting of soft tissues, when Mr. Stirling invented such an instrument. Mr. Coghill having borrowed the same instrument, passed it round for inspection. The essayist then went on to describe the various kinds of microtomes invented up to the present time. The various hardening fluids were then discussed, and the different methods of their preparation mentioned. The various modes of embedding and cutting sections were practically considered. The essay was made most interesting from the fact that Mr. Coghill kindly sent round his valuable collection of microtomes for the members to examine. The remainder of the time was occupied by the demonstration of pleuro, broncho and catarrhal pneumonia, tuberculosis, and glanders, by means of slides and the lantern, at the conclusion of which a most hearty vote of thanks was given to Mr. Coghill for his most useful and interesting paper. Before the meeting terminated Professor Penberthy proposed that "A letter of condolence be forwarded from this association to Mrs. Steel on the great loss she has sustained by the death of her husband, the late Professor J. H. Steel." Professor Axe, in seconding the resolution, spoke most feelingly of the late Professor. He had known him as a student, then as demonstrator of anatomy, and then as professor of anatomy, and never had he seen one rise to such distinction and honour so rapidly. He regarded his loss as nothing short of a calamity to the profession at large. The proposition was unanimously carried, and in silence. A vote of thanks to the Chairman brought the proceedings to a close.

### ATHLETICS.

#### Football.

INTER-HOSPITAL CHALLENGE CUP (Semi-final round).—*St. Thomas's Hospital* (Holders) *v. Middlesex Hospital.*—This match was played on March 4th, at the Richmond Athletic Grounds, Richmond, in the presence of a numerous company, St. Thomas's opposing Middlesex in the semi-final tie for the Hospitals' Challenge Cup. At twenty minutes past three Middlesex kicked off from the goal nearest the entrance end of the ground, and play for a short time was in the Saints' quarters. The Middlesex forwards obtained possession, and passed to Marshall, who made a magnificent run and succeeded in getting in. Cookson took a difficult place-kick, but failed to get it over. Shortly before half-time, White, of St. Thomas, hurt his knee and had to be carried from the ground. Praine then went back, whilst Graham played three quarter back, Turner taking his place at back. No further scoring took place, and at half time Middlesex was leading by one try to nothing. After changing ends Middlesex was compelled to touch down. Following this, St. Thomas's men made a combined attack, and Goodhue got possession and made a good run, almost getting in. The Middlesex forwards, however, playing well together, took the ball back again to St. Thomas's 25. The latter again pressed, and got very close to their opponents' goal. Five minutes before closing time the ball was passed to Praine, who, in turn, passed to Bromet, and the latter, sweeping all before him, got in amidst great cheering. Neither side scored anything further, and the game ended in a draw—one try each.

ASSOCIATION RULES.

FEBRUARY 14TH.

*Cambridge University* beat *Suffolk*, at Ipswich, two goals to nil. *St. Bartholomew's Hospital* were beaten by *Croydon*, at Croydon, by one goal to nil. *St. Bartholomew's Hospital* and *Millwall Athletic*, at Leyton, drawn, nil. *Oxford University* beat *Old Etonians*, at Oxford, by three goals to one. *London Hospital* were beaten by *Bowes Park*, at Harringay, by four goals to two.

FEBRUARY 21ST.

*Cambridge University* were beaten by *Derby County* by six goals to two. *Guy's Hospital* beat *Watford Rovers*, at Watford, by four goals to nil. *Oxford University* and *Caledonians*, at Dulwich, drawn, two goals to two. *St. Bartholomew's Hospital* beat *Millwall Athletic*, at Leyton, by two goals to nil.

FEBRUARY 28TH.

*Oxford* beat *Cambridge*, at Queen's Club, by two goals to one. *St. Bartholomew's Hospital* and *Woodville*, at Upton, drawn, one goal to one.

RUGBY RULES.

FEBRUARY 14th.

*Guy's Hospital* were beaten by *Clapham Rovers*, at Wandsworth Common by four goals one try to nil. *University College Hospital* were beaten by *Hampstead*, at Tottenham, by one goal one try to nil. *Oxford University* beat *Richmond*, at Richmond, by two goals two tries to nil. *St. George's Hospital* beat *Christ's Hospital*, at Balham, by two goals two tries to one try. *St. Thomas's Hospital* beat *Harlequins*, at Chiswich, by four goals one try to nil. *Charing Cross Hospital* were beaten by *Thurlow Park*, at Raynes Park, by three goals to nil. *St. Bartholomew's Hospital* were beaten by *Croydon* by one goal to nil. *Middlesex Hospital* beat *Saracens* at Walthamstow, by two goals two tries to nil.

FEBRUARY 27TH.

*Guy's Hospital* were beaten by *Royal Military College*, at Sandhurst, by five goals to nil. *St. Thomas's Hospital* beat *Old Merchant Taylors*, at Willesden. *University College Hospital* (second) were beaten by *St. Barnabas*, by one try to four minors. *St. Bartholomew's Hospital* beat *Marlborough Nomads*, at Surbiton, by two goals and one try to one goal and one try. *Westminster Hospital* beat *Eltham House*, by one goal and try to one try.

